# A review of selective indoor residual spraying for malaria control

**DOI:** 10.1186/s12936-024-05053-3

**Published:** 2024-08-23

**Authors:** Seth R. Irish, Derric Nimmo, Jameel Bharmel, Frederic Tripet, Pie Müller, Pablo Manrique-Saide, Sarah J. Moore

**Affiliations:** 1 Swiss Tropical and Public Health Institute, Kreuzstrasse 2, 4123 Allschwil, Switzerland; 2 University of Basel, Petersplatz 1, 4001 Basel, Switzerland; 3 Vector Control Product Testing Unit, Ifakara Health Institute, P.O. Box 74, Bagamoyo, Tanzania; 4 IVCC, Liverpool School of Tropical Medicine, Pembroke Place, Liverpool, L3 5QA UK; 5 Unidad Colaborativa para Bioensayos Entomológicos, Campus de Ciencias Biológicas y Agropecuarias, Universidad Autónoma de Yucatán, Mérida, Yucatán México; 6 Nelson Mandela African Institute of Science and Technology (NM-AIST), P.O. Box 447, Arusha, Tanzania

**Keywords:** Selective spraying, Partial spraying, Indoor residual spraying, Resting, Anopheles, Malaria

## Abstract

**Background:**

Indoor residual spraying (IRS) is one of the most effective malaria control tools. However, its application has become limited to specific contexts due to the increased costs of IRS products and implementation programmes. Selective spraying—selective spray targeted to particular areas/surfaces of dwellings—has been proposed to maintain the malaria control and resistance-management benefits of IRS while decreasing the costs of the intervention.

**Methods:**

A literature search was conducted to find (1) studies that assessed the resting behaviour of *Anopheles* mosquitoes and (2) studies that evaluated the impact of selective spraying on entomological and malaria outcomes. Additional articles were identified through hand searches of all references cited in articles identified through the initial search. A cost model was developed from PMI VectorLink IRS country programmes, and comparative cost analysis reports to describe the overall cost benefits of selective IRS.

**Results:**

In some studies, there appeared to be a clear resting preference for certain *Anopheles* species in terms of the height at which they rested. However, for other species, and particularly the major African malaria vectors, a clear resting pattern was not detected. Furthermore, resting behaviour was not measured in a standardized way.

For the selective spray studies that were assessed, there was a wide range of spray configurations, which complicates the comparison of methods. Many of these spray techniques were effective and resulted in reported 25–68% cost savings and reduced use of insecticide. The reported cost savings in the literature do not always consider all of the IRS implementation costs. Using the IRS cost model, these savings ranged from 17 to 29% for programs that targeted *Anopheles* spp. and 18–41% for programmes that targeted *Aedes aegypti*.

**Conclusions:**

Resting behaviour is generally measured in a simplistic way; noting the resting spot of mosquitoes in the morning. This is likely an oversimplification, and there is a need for better monitoring of resting mosquitoes. This may improve the target surface for selective spray techniques, which could reduce the cost of IRS while maintaining its effectiveness. Reporting of cost savings should be calculated considering the entire implementation costs, and a cost model was provided for future calculations.

## Background

Malaria continues to cause high levels of morbidity and mortality, particularly in Africa, where the majority of malaria cases occur [1]. In 2022, malaria cases increased to an estimated 249 million cases, resulting in an estimated 608,000 deaths [1]. To decrease the number of cases, it is important to invest in effective testing and treatment of malaria, as well as undertaking strategies that prevent malaria transmission. Vector control is the most effective current malaria prevention strategy, and the main techniques employed are the distribution of insecticide-treated nets (ITNs) and indoor residual spraying (IRS). In recent years, there has been the development of highly effective nets with different active ingredients (e.g. [2, 3]). This has resulted in some countries stopping their IRS programs, partly due to cost considerations, even though IRS remains highly cost effective [4, 5]. However, IRS has several advantages which might be useful if the costs of IRS could be reduced. These advantages include the possibility for insecticide rotation as part of a resistance management plan [6], less necessity for active utilization (as compared to ITNs, which must be put in place by homeowners each night) [7], and, similar to ITNs, IRS can have a community protection effect when coverage is high [8].

One way to decrease the cost of IRS is through selective indoor spraying of some of the surfaces in houses. It should be noted that selective spraying is sometimes termed “targeted IRS” [9] or “partial IRS” [10] that should be distinguished from the targeted application of IRS to areas where there is evidence of recent malaria transmission rather than blanket application to all houses [11]. Conventional IRS recommended by the WHO for malaria control [12] involves the full spraying of all indoor walls and often the ceilings of houses.

Optimally, the selective indoor spray is applied where mosquitoes are most likely to rest [12]. Selectively applying residual insecticides, e.g., for *Aedes aegypti* on exposed lower sections of walls (< 1.5 m), under furniture, and on dark surfaces throughout houses provides an entomological impact similar to spraying entire walls (as performed in classic IRS), but in a fraction of the time (< 18%) and insecticide volume (< 30%) compared to classic IRS [9]. Other studies have shown important impacts using selective spraying [13, 14]. This selective spraying approach is endorsed by the Pan American Health Organization for IRS spraying for control of *Aedes aegypti* in urban settings [15]. While numerous studies have been done to evaluate selective IRS for malaria control, this work has not provided conclusive findings required to change current policies. This narrative review summarizes previous research on the use of selective spraying for vector-borne disease control and the cost-saving implications to see whether there might be justification for the use of selective spraying for malaria control, and to determine what avenues of research might be the most impactful to maximize its efficacy.

## Methods

### Selection criteria

Studies were included if they considered the two key questions of this review: resting behaviour of *Anopheles* mosquitoes or efficacy of selective indoor residual spraying.

### Search strategy

An initial search was conducted on PubMed in July 2022, without language or date limits to find (1) studies that assessed the resting behaviour of *Anopheles* mosquitoes and (2) studies that evaluated the impact of selective spraying on entomological and malaria outcomes. Search terms included “partial indoor residual spraying” and “targeted indoor residual spraying”. Additional articles were identified through hand searches of all references cited in articles identified through the initial search. This process continued until no further related articles were found.

### Data extraction

Data from the selected papers were extracted to determine the resting heights and behaviours of *Anopheles* mosquitoes. Additionally, data was extracted from articles that discussed the impact of selective spraying, and the impact and cost savings of these studies were summarized.

### Cost analysis

A cost model was constructed from the PMI VectorLink IRS country programs comparative cost analysis reports. The model is based mainly on the 2018 data across the 14 countries where PMI VectorLink performed IRS [16]. A comparison to the cost analysis data from 2019 to 2022 shows that the relative cost breakdown for each area has not changed significantly. The spray campaign costs were broken down further using the following data and assumptions. Training costs were calculated from the average percentage spray campaign costs used for Malawi, Rwanda and Uganda for training of trainers and SOP and team leader training (data provided by PMI VectorLink). Spray campaign personnel costs were calculated from the total campaign days and the daily wages minus the training costs. The rest of the spray campaign costs were assigned to transportation of spray personnel (mainly vehicle hire, drivers and fuel).

## Results

The main results from this review were separated into two categories, (1) description of the resting sites of mosquitoes inside houses and (2) reports of experiments or operational pilots of selective spraying. Seventeen studies were found reporting the resting sites of mosquitoes in houses, and nine were found reporting on experiments or pilot studies of selective spraying.

### Resting sites of mosquitoes in houses

#### Resting height

The results collated from the reviewed publications showed clear evidence that the resting sites and behaviour of the mosquitoes vary. These variations were observed both between and occasionally within species. In many of the publications, the height (distance above the floor) at which mosquitoes were collected was reported.

Based on these data, it was determined that *Anopheles darlingi*, *Anopheles aquasalis, Anopheles ludlowi, Anopheles hyrcanus, Anopheles fluviatilis, Anopheles leucosphyrus, Anopheles aconitus, Anopheles kochi, Anopheles subpictus, Anopheles indefinitus, Anopheles marajoara*, *Anopheles punctimacula, Anopheles nuneztovari*, and *Anopheles flavirostris* tended to rest primarily on the lower half of walls [17–23].

In contrast, *Anopheles barbirostris*, *Anopheles oswaldi*, and *Anopheles rangeli* were found to rest above 1.5 m above the floor, and often higher [21, 22]. Sahu et al. [24] found 99% of *Anopheles minimus* and *Anopheles fluviatilis* to rest on walls (as opposed to eaves, hanging objects, and the roof), with most of these mosquitoes resting between 90 and 125 cm from the ground.

It is important to note that most of these studies were conducted outside of Africa. Despite this, a few key studies based in Africa have investigated the resting behaviour of *Anopheles gambiae *sensu lato (*s.l.*) and *Anopheles funestus* vectors*.* These studies can largely be grouped into monitoring the height of the resting site on the wall or roof, additional observations about the substrate on which mosquitoes rest, and their resting behaviour conducted within experimental huts were also noted.

In his first study looking at the resting height of malarial vectors, Smith [25]investigated the distribution of *An. gambiae* and *An. funestus* vectors in cone huts on Ukara Island (a Tanzanian island in Lake Victoria, near Mwanza). These cone huts measured 6.4 m high and 6.9 m wide at their bases, and typically housed both humans and cattle. The huts were searched until all observable mosquitoes had been collected and their location of collection was recorded. From the trial it was shown that the vast majority of female mosquitoes (80% of *An. gambiae* and 79% of *An. funestus*) were found to be resting below 2.1 m (from the floor) in the huts during the rainy season. The majority of these rested on the human-habited side of the huts; nevertheless, considerable numbers were also found on the cattle-habited side of the huts. The same trend was found during the dry season. Later, Smith [26] collected mosquitoes from houses of three different types (*tembe*, *msonge*, and *banda*) in Tanzania. Initial catches were conducted between 0800 and 1200 with additional complementary catches between 1100 and 1500 being conducted three days later. During the collection period, the proportion of *An. gambiae* mosquitoes resting on the roof ranged from 42 to 74%. There were no large differences between the proportions resting on the roof during the night and day, but there were differences in roof-resting between the different types of huts. Mathis et al. [27] reported 94.6% of *An. gambiae* and *An. funestus* were collected on the ceilings in monitored huts. On the contrary, Mutinga et al. [28] noted *An. gambiae* mosquitoes resting primarily on the lower parts of walls and the darker parts of the room. Osae [29] found large proportions of all three species resting above 2 m (*An. gambiae*: 76%, *Anopheles coluzzii* 58%, *An. funestus* 74%), and preferably on dark materials in cool, humid areas. Sande et al. [30] found the highest proportion of *An. funestus* and *An. gambiae* on the roof (although considerable numbers were found on walls, with fewer mosquitoes collected on furniture. When only wall surfaces were considered, the majority were collected below 1 m (44% of *An. funestus*, 64% of *An. gambiae s.l.*). Msugupakulya et al. [31] evaluated the resting sites of *An. gambiae* and *An. funestus* in different types of houses. They found that the highest numbers of mosquitoes rested on the roof in houses with thatched roofs (with the exception of *An. funestus* in brick houses), and in houses with metal roofs, the highest numbers of mosquitoes rested on surfaces other than walls or roofs. It is worth noting that in all types of houses, mosquitoes were found resting on walls, roofs, and other surfaces (Table 1).

**Table 1:** Studies evaluating the resting sites of major African malaria vectors in houses

References	Country	Species	Type of house	Percentage on walls	Percentage on roofs	Percentage on other
Osae 2014	Ghana	*An. gambiae*	Mud, brick, and cement houses with tile or metal roofs	9	56	35
Sande et al. 2016	Zimbabwe		Mud, brick, and cement houses with tile or metal roofs	36	42	16
Coleman et al. 2021	Ghana		Experimental hut, tarpaulin ceiling	51	45	5
			MEAN	32	48	19
Osae 2014	Ghana	*An. coluzzii*	Mud, brick, and cement houses with tile or metal roofs	29	25	45
Chabi et al. 2023	Côte d'Ivoire		Experimental hut, plywood ceiling	63	31	6
			MEAN	46	28	26
Osae 2014	Ghana	*An. funestus*	Mud, brick, and cement houses with tile or metal roofs	23	59	18
Sande et al. 2016	Zimbabwe		Mud, brick, and cement houses with tile or metal roofs	40	50	7
Msugupakulya et al. 2020	Tanzania		Thatched roofs and mud walls, no ceiliings	18	55	27
Msugupakulya et al. 2020	Tanzania		Thatched roofs and brick walls, no ceilings	25	33	43
Msugupakulya et al. 2020	Tanzania		Metal roofs and unplastered brick walls, no ceilings	37	16	47
Msugupakulya et al. 2020	Tanzania		Metal roofs and plastered brick walls, no ceilings	27	20	53
			MEAN	28	39	32
Msugupakulya et al. 2020	Tanzania	*An. arabiensis*	Thatched roofs and mud walls	21	43	36
Msugupakulya et al. 2020	Tanzania		Thatched roofs and brick walls	13	50	38
Msugupakulya et al. 2020	Tanzania		Metal roofs and unplastered brick walls	27	8	66
Msugupakulya et al. 2020	Tanzania		Metal roofs and plastered brick walls	10	30	60
			MEAN	18	33	50

#### Resting substrate

Other studies have looked at the effect of resting substrate or other factors on the resting behaviour of African malaria vectors. Smith [26] evaluated the impact of different factors within experimental huts to evaluate their impact on mosquito resting behaviour. He found that neither building a partition wall in the hut, modifying the hut entry site, adding a ceiling, modifying the surface of the roof, nor the abdominal status (or source of blood meal) appeared to change the resting behaviour of *An. gambiae* in terms of resting on the roof or walls. However, modifying the substrate of the walls (from smooth mud to rough mud) resulted in greater resting on rough mud walls. Similarly, making a fire inside the huts resulted in decreased resting on the roof and increased resting on walls. Beds were not a major resting site for mosquitoes in experimental huts, with only nine percent of mosquitoes collected from beds. Mutinga et al. [28] found that *An. gambiae* preferred to rest on fabric attached to the walls. Osae found differences in resting sites between *An. gambiae*, *An. coluzzii*, and *An. funestus* in Ghana [29]. He found the main resting sites to be roofing beams for *An. gambiae* (28%), on netting or frames of windows for *An. coluzzii* (20%), and for *An. funestus,* it was the roof. He also looked at the materials that mosquitoes were resting, with *An. gambiae* and *An. funestus* resting primarily on wood surfaces, and *An. coluzzii* resting on nylon.

### Resting sites of mosquitoes in experimental huts

Finally, some studies taking place in experimental huts have monitored the resting behaviour prior to introducing interventions such as wall spraying. Smith [26] found higher proportions of *An. gambiae* resting on the roof in experimental huts than in other types of structures, with 94–97% of mosquitoes resting on roofs, compared with 42–74% in local houses. Coleman et al. [10] monitored the resting sites of *An. gambiae s.l.* collected in West African experimental huts in Ghana. The majority of *An. gambiae s.l.* were collected from the ceiling and the top half of the veranda. In a follow up study, Chabi et al. [32] found 43% of *An. gambiae s.l.* resting on the lower half of walls, 24% of mosquitoes resting on the top half of walls, and 33% of mosquitoes resting on ceiling.

### Evaluation of selective spraying

In the first year of the “Sardinian Project” an attempt to eliminate *Anopheles labranchiae* from Sardinia, selective spraying was conducted with spraying of walls below 1.5 m in the first campaign (1946–1947), but in successive campaigns “full spraying” was conducted [33]. Malaria cases declined from 74,641 in the first year (1946) to 39,303 in the second [34], although the impact of selective spraying with DDT cannot be disentangled from the impact of large-scale aerial adulticide/larvicide application and source reduction that was carried out in parallel. This highlights the previous/historical use of selective IRS, however, no further details on impact of the intervention were provided in this source.

Another method of selective spraying was evaluated in Lebanon [35], where “band spraying” was attempted, spraying horizontal swaths of DDT of 30 cm width separated by an equal distance of unsprayed areas (all 1 m above the ground). The impact of this type of spraying was measured in areas where *Anopheles sacharovi* and *Anopheles superpictus* were the main vectors both by looking at malaria rates, and collection of *Anopheles* in houses in areas where full spraying or selective spraying had been conducted (relative to control areas). While no impact on parasite rates was found, due to a drop in cases in both control and treatment areas, there was a reduction in *Anopheles* in the full and selectively sprayed houses. The authors estimated the cost savings that might be found with selective spraying was approximately 31.3% (including the costs of DDT, labour, transport, and storage (Table 2).

**Table 2:** The impact and cost savings of selective spraying studies

Study	Location	Main vector(s)	Insecticide	Type of selective spraying	Impact of selective spraying compared with full spraying	Reported cost savings with selective spraying (%)	Reduction in insecticide use (%)	Based on the reduction in days required to spray an equivalent number of houses		Overall reduction in total operational costs (cost model) (%)
								Reduction in spray team wages/meals (%)	Reduction in transport costs (%)	
Gramiccia et al. 1953	Lebanon	*An. sacharovi, An. superpictus*	DDT	Horizontal band spraying	Difficult to determine, substantial decrease in all arms (including control)	31.3	35.8	26.3	21.7	15.4
Pletsch & Demos 1954	Taiwan	*An. minimus*	DDT	Focusing spraying on sleeping rooms	After two rounds of spray, infant parasite rates were 0% in both arms	25.6	38.4	–	–	–
Lassen et al. 1972	El Salvador	*An. albimanus*	Propoxur	Two swaths on the inner side of the roof and in the angle between the roof and the wall	Full spraying not evaluated, but ~ 10% of malaria prevalence in selective spray area compared to control area	50–60	–	–	–	–
Gandahusada et al. 1984	Indonesia	*An. aconitus*	fenitrothion	Sprayed between 10 and 85 cm	Substantial decrease in both arms, but more impact on *P. falciparum* with full spraying	68.0	64.5	46.0	46.0^a^	28.6
Asinas et al. 1994	Philippines	*An. flavirostris*	bendiocarb	Experimental huts sprayed up to 0.7 m on the walls, and all areas within 0.1 m of the door, window, and internal and external eaves	Never more than 8% difference in mosquito mortality compared to the fully sprayed hut	36% spray time, 49% less insecticide	49.0	36.0	36.0^a^	21.9
Arredondo-Jimenez et al. 1995	Mexico	*An. albimaus*	bendiocarb	Experimental huts sprayed with two 1 m horizontal swaths, one from 0.75–1.75 cm on the walls, and one on the roof, starting at its intersection with the wall	No significant difference in mortality between fully sprayed and selective sprayed huts	40.0	40.5^b^	38.5^b^	38.5^a^	19.5
Dunbar et al. [45]	Mexico	*Ae. aegypti*	bendiocarb	Experimental houses sprayed on walls below 1.5 m and under furniture (targeted IRS, TIRS) or under furniture only (resting site targeted IRS, RS-TIRS)	No difference in first two months for RS-TIRS, or for first 4 months with TIRS	TIRS	38.0	31.0	31.0^a^	17.5
						RS-IRS	85.0	82.0	82.0^a^	41.1
Coleman et al. 2021	Ghana	*An. gambiae s.l*	pirimiphos-methyl	Experimental huts sprayed with bottom half, bottom half + ceiling, top half, top half + ceiling, or full spray. A field study that compared full spraying, selective spraying (top half + ceiling), and no spray	No significant differences in mortality or human biting rates between fully and selectively sprayed huts/houses. Both were better than control	36.0	39.2	25.7	25.7	17.0

^a^extrapolated from percentage saving in spray team wages/time

^b^extrapolated from Table 3 in the publication

Pletsch and Demos [36] reported “selective spraying” in Taiwan against *Anopheles minimus*. Full spraying was conducted by spraying walls, roofs, ceilings, and undersides of furniture with DDT (2 g/m^2^). The inner walls and undersides of roofs of all outbuildings were also sprayed except for the first 50 cm of the wall in pig pens. “Selective spraying” was done in several ways; on the walls of bedrooms and storerooms, the underside of the roof in bedrooms, the ceilings in bedrooms and storerooms (which were quite rare), the undersides of furniture and window recesses in bedrooms, storerooms, sitting rooms, and kitchens (only inside and under the food cabinet), and the underside of the bed or bed platform in bedrooms. Any room in which people slept was considered a bedroom. The results from two rounds of both spray types were positive, reducing malaria rates from over 20% to less than 1% in Chi-Shan, and reducing them from about 2% to 0% in an additional study in central Taiwan. Both entomological investigations supported the finding of effective control and reduction of the numbers of mosquitoes collected in bedrooms to zero with both techniques. The cost savings were generated from spraying 38.4% less surface area in the selective spraying treatment, and the overall costs were reduced by 25.6%. However, some disadvantages of selective spraying were noted, specifically, the detection of *An. minimus* mosquitoes in cattle sheds (a possible harborage that could result in the build-up of resistance), the detection of *Anopheles sinensis* in cattle sheds that bothered the farmers’ water buffalo, and hesitation from homeowners and sprayers about receiving less than full coverage.

Gandahusada et al. [37] built on the knowledge about *Anopheles aconitus* resting sites to evaluate full and selective spraying in Java, Indonesia, using fenitrothion as *An. aconitus* populations were becoming resistant to DDT. They designed three areas for the study, one for full spraying, one for selective spraying (between 10 and 85 cm on the wall, in addition to full spraying of cattle shelters), and one for the control. Cholinesterase levels were monitored in the sprayers to prevent negative health effects from exposure to the insecticide. More sprayers in the full spray arm had > 50% reduction in cholinesterase than those in the selective spray arm, indicating less exposure for those conducting the selective spray. The full spray arm reduced malaria slide-positive rates from 6.5% to 0.4%, while selective spray reduced the rate from 1.9% to 0.3%. However, there was a more substantial decrease in the *Plasmodium falciparum* index (proportion of cases caused by *P. falciparum*) in the full coverage area than in the selective spray area.

Asinas et al. [23] observed resting heights of *Anopheles flavirostris* in a site outside of Manila, Philippines. They found the vast majority resting below 1 m on the walls and evaluated the impact of selective spraying (the lower 70 cm of the wall, as well as 10 cm around windows and interior and exterior eaves) in experimental huts for 6 months. They found similar results for full spraying and selective spraying, with never more than an 8% difference in mosquito mortality between the two.

Arredondo Jiménez et al. [38] evaluated full spraying and selective spraying (a horizontal swath on the wall between 0.75 and 1.75 m from the floor, as well as a 1 m swath of the roof from where it met the wall) with bendiocarb in Mexico. They followed the community for two years (over four spray rounds) and measured the entomological impact. They did not note substantial differences between the fully sprayed and selectively sprayed areas in terms of residual activity of the insecticide, resting behaviour or mortality of *An. albimanus* mosquitoes, or human landing collections. They found a 50% savings in spraying time in the selective spray area and 40% overall cost savings.

Coleman et al. [10] conducted an experimental hut study coupled with a village-level study to evaluate selective spraying. The experimental hut study evaluated half walls (lower and upper) in combination with the ceiling with full spraying. There was no significant difference in mortality of *An. gambiae s.l.* found between full spraying and either of the selective spraying treatments. The inclusion of the ceiling appeared to be important, as the mortality was more than 20% higher when the ceiling was included in the treatment arms. There was also no significant difference between human biting rates between full and selectively sprayed communities (upper half + ceiling), and both were significantly lower than in unsprayed communities.

Chabi et al. [32] conducted an experimental hut trial in Côte d’Ivoire, in an area of intense pyrethroid resistance. Three IRS insecticides (pirimiphos methyl 1 g/m^2^ (Actellic), clothianidin 300 mg/m^2^ (SumiShield) and clothianidin 200 mg/m^2^ + deltamethrin 25 mg/m^2^(Fludora Fusion)) were evaluated with four treatments (unsprayed, fully sprayed, bottom half of the wall + ceiling, upper half of wall + ceiling). For all three insecticides, there was slightly higher mortality with the bottom half of the wall + ceiling than the upper half of the wall + ceiling. The differences in mortality between full spray and the two selective spray treatments were not statistically significant except for clothianidin, where the top half + ceiling spray resulted in less mortality than the other two treatments.

Snetselaar et al. (pers. commun.) evaluated selective spraying and uneven spraying in release-recapture and experimental hut studies. In the release-recapture study, *Anopheles gambiae* Kisumu (susceptible to all insecticides tested) was released in huts with clothianidin 200 mg/m2 + deltamethrin 25 mg/m2 (Fludora Fusion) sprayed using a selective, checkerboard spray (50% of walls sprayed), uneven spray (some areas sprayed at 10%, others at 100%, and others at 190%), full spray (manual or with a track sprayer), as well as full spraying of pirimithos-methyl 1 g/m^2^ (Actellic). Mortality (24 h) was not significantly different between any of the treatments. For the experimental hut trial with *Anopheles arabiensis*, the highest 24 h mortality was found with the track sprayer full spray, and the mortality was not significantly different between the other treatments.

### IRS program cost analysis

**Fig. 1:**
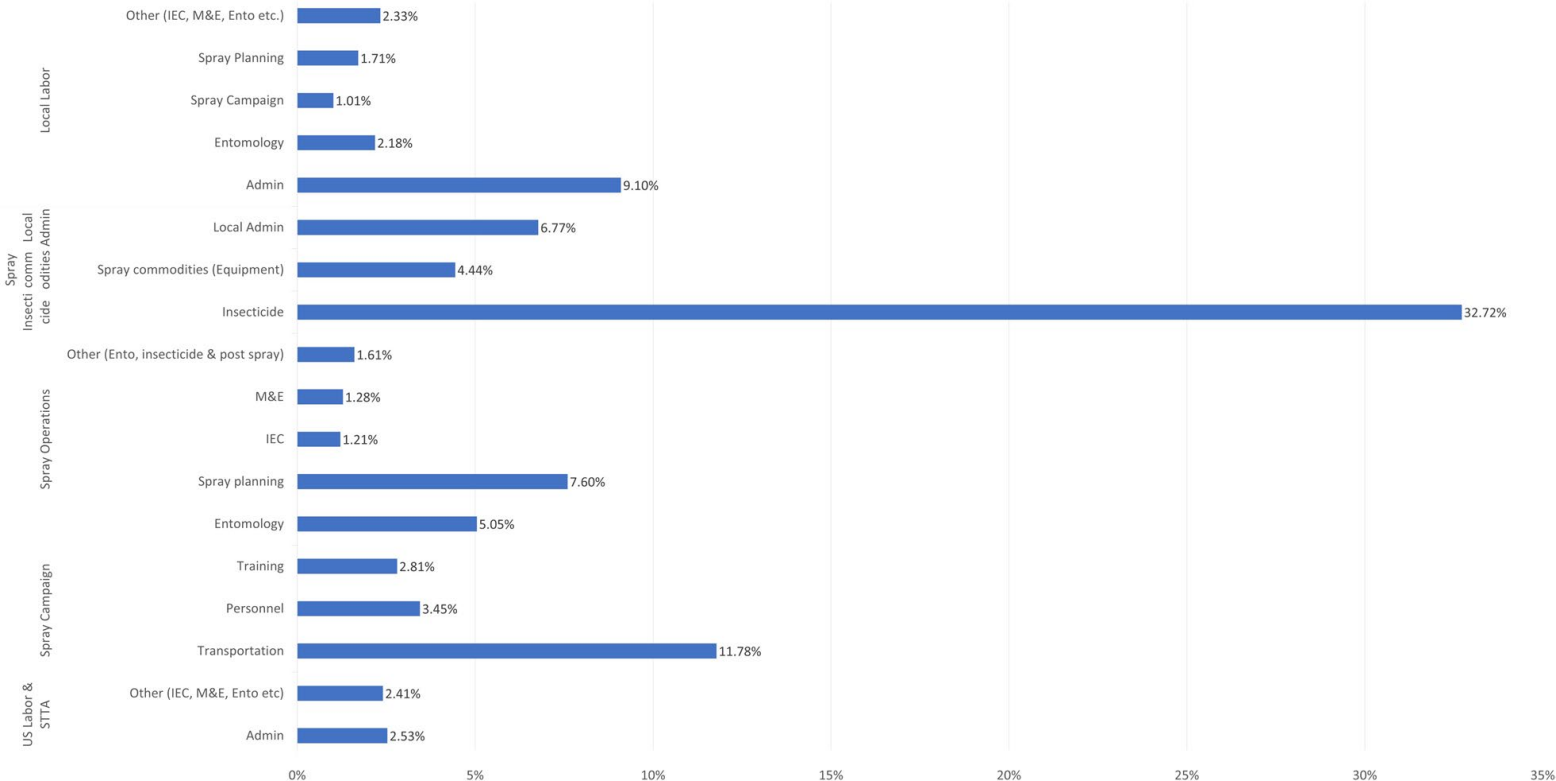
Percentage breakdown of the average PMI VectorLink IRS spray campaign programme costs (2018–2021)

The percentage break down of the PMI VectorLink IRS program costs are shown in Fig. 1.

For all of the publications where cost savings of IRS are reported, most authors have shown the data for reduction in insecticide use and spray team costs (mainly staff costs) (Table 2). Coleman et al. [10] have also extrapolated that a reduction in the spray time (due to decreased spraying and non-removal of items from the house) would also reduce the transportation costs by 26%, as the team could spray more houses in a day, requiring less travel to complete the same number of houses.

**Fig. 2:**
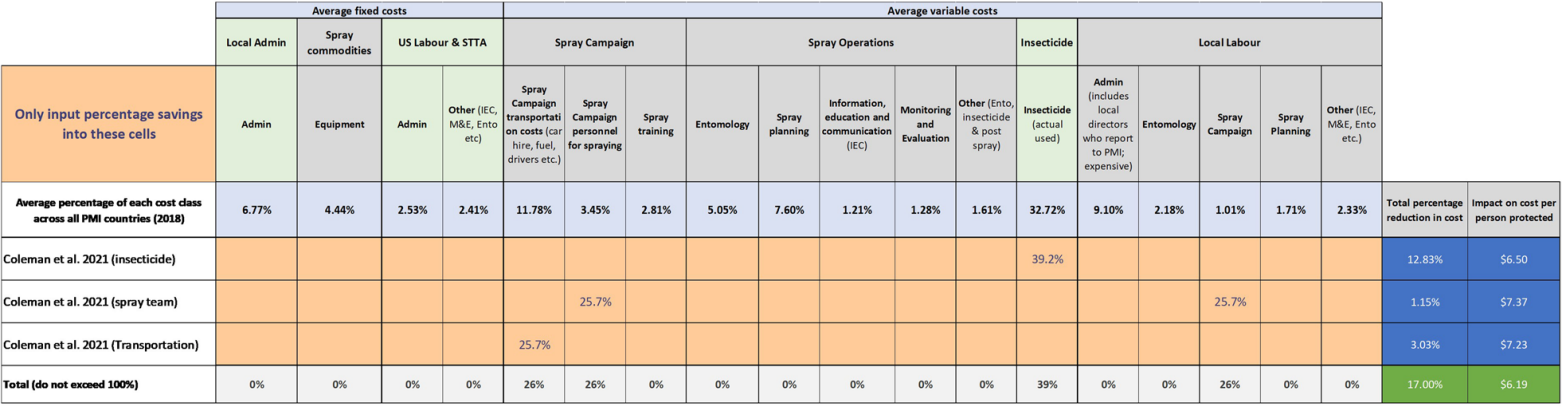
An example of the cost model inputs and outputs

Using these assumptions, the IRS cost model was used to show the overall savings that could be achieved when the entire programme costs are included, such as administration, monitoring, entomology, and community engagement. An example of the inputs and outputs from the IRS cost model are shown in Fig. 2 for the results reported by Coleman et al. [10]. The overall savings when selective IRS was used ranged from 15.5 to 28.6% for programmes which targeted *Anopheles* mosquitoes and 17.5–41.1% for programs that targeted *Ae. aegypti*.

## Discussion

Selective spraying has been repeatedly proposed as a solution to optimize the cost effectiveness and minimize the logistical challenges of IRS. Observations of patterns in the resting behaviour of mosquitoes have led to the conclusion that if preferred resting places are sprayed, then a comparable impact can be achieved with less (but more targeted) spraying. As several authors have noted, this depends on using a non-irritant insecticide to ensure that mosquitoes do not avoid sprayed areas [23, 39].

From some of the operational pilots and experimental hut studies, it appears that selective spraying can result in comparable results at a reduced cost. Some studies noted epidemiological impacts at reduced costs [36, 37, 40], whereas other studies noted important entomological impacts [10, 23, 32, 35, 38]. In some cases, there appeared to be a slightly reduced effect or other disadvantages such as possible selection of resistance, slower rates of decrease in malaria rates, and reluctance from homeowners [36, 37], whereas in other cases, there appeared to be advantages other than reduced costs, i.e. reduced insecticide exposure [37].

An essential part of selective spraying is the determination of what parts of houses should be sprayed and what parts of houses should not be sprayed. While in some cases, this decision has been informed by previous work, in other cases, the choice seems to be somewhat arbitrary. The two main factors that could inform selective spraying are logistical (i.e. making spraying houses easier and faster) or behavioural (using the behaviour of the mosquito to target the key resting spaces).

Aspects of spraying that would reduce the amount of spraying and logistical costs could include:

- Spraying that can be done from outside houses (including eaves, animal shelters)
- Spraying that does not require the movement of furniture (upper halves of walls, ceilings, undersides of furniture), which may additionally benefit from increased user uptake
- Targeted spraying of houses (i.e., only spraying houses at the edges of a village, near breeding sites or houses with children under five years of age)

Selective spraying might be improved through improved monitoring of the resting behaviour of mosquitoes through careful recording of:

- Rooms in which mosquitoes are resting (bedrooms, kitchens, bathrooms, animal shelters)
- The height of resting sites on the wall
- The type of building in which mosquitoes are resting
- The amount of light (lux) present in resting site
- Temperature and humidity of resting sites
- Air movements
- The substrate on which mosquitoes are resting (wood, mud, clothes, furniture)(see Table 3 in [41])
- The interaction between an insecticide and a mosquito (toxicity and irritancy)
- Resting behaviour related to seasonality [25]
- Types of houses (wall substrate, roof material) [31]
- Orientation (north, east, west, south) with respect to sun, climatic conditions
- Resting behaviour of mosquitoes infected with *Plasmodium* parasites.

As seen above, the behaviour of mosquitoes (in combination with an understanding of logistical issues) is essential for understanding the optimal design of a selective spray programme. One of the challenges for understanding the resting behaviour of mosquitoes is the fact that mosquitoes may move around the inside of houses over the course of the night, but the collection of mosquitoes at dawn may only capture one aspect of this movement. Indeed, when mosquitoes have been collected at different times or monitored through observation, it has been shown that they are moving inside houses to some degree [26, 42]. It is likely that mosquitoes balance the need for homeostasis (optimal temperature and humidity) [43] with a choice of colours and low light to be the least visible. Better methods for monitoring mosquitoes (video recording, motion sensing, collections at multiple times) may allow for better targeting of insecticides. Furthermore, when multiple vector species are present in the same location, the behaviour of both must be considered when targeting insecticide spray.

This improved monitoring of resting site behaviour would seem especially important for the major African malaria vectors, *An. gambiae s.l.* and *An. funestus*, as there appear to be contradictory findings in the literature. The earliest recording of resting heights found most *An. gambiae* and *An. funestus* to be resting on walls below 2.1 m; however, this was in “cone huts” that reached 6.4 m in height [25]. Mathis et al. [27] reported that 94.6% of *An. gambiae* and *An. funestus* collected in houses were resting on the ceiling. Mutinga et al. [28] stated that *An. gambiae* rested primarily on the lower parts of walls, on fabric, and on the dark side of the room. Osae [29] reported a number of resting sites for *An. gambiae*, *An. coluzzii*, and *An. funestus*. He stated that most of the *An. gambiae* (56%) and *An. funestus* (59%) were resting on roofs, roofing beams, and ceilings between 6:00 and 10:00, whereas only 25% of *An. coluzzii* were found there. Msugupakulya et al. [31] found very low numbers of *An. funestus* (16–20%) and *An. arabiensis* (8–30%) resting under metal roofs, although higher numbers of the two species when roofs were thatched (*An. funestus* (33–55%), *An. arabiensis* (43–50%)). Importantly, they noted that considerable proportions of mosquitoes in all houses were resting on “other surfaces” than walls and roofs, presenting challenges for spraying (although the movement in houses is not to be forgotten). The two most recent experimental hut studies found different results in their pre-spray collections, with the majority of *An. gambiae s.s.* remaining in huts in northern Ghana being found on the ceiling (followed by the top half of the wall), whereas the *An. coluzzii* in Côte d’Ivoire were primarily resting on the bottom half of the wall (followed by the ceiling). The apparent difference in behaviour might explain why in Côte d’Ivoire, in huts treated with clothianidin, the bottom half + ceiling treatment was more effective than the top half + ceiling treatment. However, there is much to be learned about the behaviour of mosquitoes inside houses, and what to do when there are multiple vector species. A better understanding of this behaviour will allow the development of better selective spray methods.

The potential cost savings of selective IRS could be substantial; and reported savings in the literature range from 38 to 85% for insecticide use and 25.7–82% for spray team costs (wages and food). The level of cost reduction depends on the type of selective spraying employed. In some cases, the selective spraying was limited to a single band in houses [37], whereas in other studies, it was only half of the wall that was excluded [10, 32]. Reductions in costs can come from reduced insecticide and reduced time required to treat houses, especially if furniture does not have to be removed, including spray pump refilling time and water collection. These time savings should be monitored in future studies.

However, these reported cost savings do not consider other costs typically associated with an IRS control programme, such as surveillance and monitoring, administrative staff, chemical storage, environmental assessment, equipment etc. To understand the impact of selective IRS on the total cost of an IRS programme, an IRS cost model was developed from cost analysis reports of PMI VectorLink country programmes. When considering other IRS programme costs and accounting for savings in transport costs not reported in some publications, the overall cost savings ranged from 15.5 to 28.6% for programmes targeting *Anopheles* mosquitoes*.*

These percentage cost savings could reduce the cost per person per year of a PMI VectorLink IRS programme from USD 7.46 (average for 2020–2022) to between USD 5.33 to USD 6.19. These represent substantial cost savings of between 17 and 29%. However, the cost of IRS programmes has substantially increased over the past five years, from USD 5.36 per person per year in 2018 to USD 7.69 in 2022, and therefore the impact is substantially reduced due to rising costs [44].

Not all IRS programmes are run as comprehensively as PMI VectorLink programmes, and they may not have all the additional costs besides transport, staff for spraying, and insecticide, which may significantly increase the relative cost advantage of selective IRS. There may be other control programmes with lower costs, but these were not identified in the current review.

## Conclusions

A clear understanding of mosquito resting behaviour is key to the effectiveness of indoor residual spraying, one of the major malaria control interventions. Currently, indoor residual spraying is conducted by spraying all sprayable interior surfaces of a house to maximize the likelihood of a mosquito coming in contact with the insecticide. However, this may not be necessary if mosquitoes preferentially rest on certain surfaces of the house. This review aimed to assess the resting behaviour of *Anopheles* mosquitoes. There were no clear patterns for African malaria vectors, and standardized methods for monitoring resting behaviour are necessary before a spray campaign is implemented. The existing data on selective spraying indicate that this may be a promising way of controlling malaria, but further work is necessary. The overall impact of selective IRS on control programme costs could be substantial, reducing the total programme costs by up to 30–40%, which could help mitigate some of the increased programme costs incurred over the past few years and help maintain IRS coverage and impact. However, these cost reductions must also be carefully considered against the total cost of an IRS programme, not just the spraying operations and insecticide costs.

IRS is being phased out from an increasing number of countries due to its cost despite clear evidence of effectiveness for malaria control and insecticide resistance management. Several operational studies have indicated substantial decreases in malaria prevalence using selective spraying at a fraction of the cost of full spraying. Studies that evaluate the entomological and epidemiological impact of selective spraying with existing IRS compounds are urgently required to enable this method to be fully validated and, if successful, pass on these cost savings to help maintain this important vector control tool.

## Data Availability

There is no new data presented here, and all can be found in published articles. Excel files for the cost model are available upon request.

## Abbreviations

DDT: Dichlorodiphenyltrichloroethane
IRS: Indoor residual spraying
ITN: Insecticide-treated Net
PAHO: Pan American health organization
WHO: World health organization
PMI: Presidents malaria initiative
